# Neonatal and Birth Risk Factors for Type 1 Diabetes Mellitus: Prediction Using an Artificial Neural Network

**DOI:** 10.3390/life15121800

**Published:** 2025-11-24

**Authors:** Claudiu Cobuz, Mădălina Ungureanu-Iuga, Maricela Cobuz

**Affiliations:** 1 Faculty of Medicine and Biological Sciences, Ştefan cel Mare University of Suceava, 13th Universităţii Street, 720229 Suceava, Romania; claudiu.cobuz@usm.ro (C.C.); cobuz.maricela@spjsv.ro (M.C.); 2 Integrated Center for Research, Development and Innovation in Advanced Materials, Nanotechnologies, and Distributed Systems for Fabrication and Control (MANSiD), Ştefan cel Mare University of Suceava, 13th Universităţii Street, 720229 Suceava, Romania; 3 ”Sfântul Ioan cel Nou” Emergency Clinical Hospital, 720224 Suceava, Romania

**Keywords:** Diabetes Mellitus, infant nutrition, birth weight, Apgar score, machine learning, multifactorial analysis

## Abstract

Type 1 Diabetes Mellitus (T1D) can be related to various factors, including neonatal and perinatal conditions. This study investigated the impact of neonatal and perinatal factors—Apgar score, birth weight, feeding type, sex, and delivery type—on the risk of Type 1 Diabetes Mellitus and evaluated predictive models. A cohort of 327 patients was analyzed using correlations, General Linear Model, and artificial neural network. T1D patients showed higher birth weight, lower Apgar score, a predominance of formula feeding, and more cesarean deliveries. Diabetes risk showed a moderate positive correlation with birth weight class and nutrition type (*p* < 0.05) and a weak negative correlation with Apgar score (*p* < 0.05), while birth weight, birth weight class, and nutrition type were all weakly positively correlated with each other and with delivery type (*p* < 0.05). General linear model identified nutrition type, birth weight, and Apgar score as key predictors, with significant interactions among them (R^2^ = 0.88). Artificial neural network achieved high accuracy (84.2%), AUC (0.95), sensitivity (89.1%), and specificity (76.7%). ANN successfully modeled the complex non-linear interactions among early-life factors, allowing it to discriminate between high-risk and low-risk cases, as evidenced by the low prediction errors (RMSE 0.31 and MAE 0.09) and strong agreement (Kappa 0.81). The models’ strong internal predictive performance points to potential applications in early T1D diagnosis and personalized management, although confirmation in larger and independent datasets is needed.

## 1. Introduction

Type 1 Diabetes Mellitus (T1D) is a chronic autoimmune disease characterized by the loss of pancreatic beta-cells, which results in the eventual lack of insulin production and subsequent hyperglycemia [1]. This condition primarily affects children and adolescents, typically presenting acutely with symptoms such as polyuria, polydipsia, weight loss, and ketoacidosis, necessitating lifelong treatment with exogenous insulin [1,2]. Globally, the incidence of T1D has been increasing rapidly over the past few decades, a trend documented across various regions, including annual increases reported in Europe and North America [3,4]. This escalating prevalence emphasizes the importance of understanding the underlying factors driving disease development.

The etiology of T1D is complex, stemming from the interaction between genetic susceptibility and environmental factors. While human leukocyte antigen complex is known to have a significant importance in the pathogenesis of T1D, environmental influences, particularly those encountered during fetal life, birth, and infancy, are crucial contributors to T1D risk [5,6]. Investigating these maternal background and perinatal factors is essential for clarifying previously reported associations and exploring new ones related to T1D risk in childhood and adolescence.

Several maternal and perinatal factors have been associated with altered T1D risk, according to observational studies and meta-analyses. Maternal diabetes, including pre-existing type 1 or type 2 diabetes before delivery, has been strongly linked to increased risk in the offspring [7]. For instance, a register-based study in Finland reported that maternal diabetes was associated with a high adjusted risk for the child (HR = 6.42; 95% CI 5.35, 7.72) [2]. This association suggests that the intrauterine environment, particularly exposure to increased delivery of nutrients like glucose and free fatty acids, and resulting enhanced fetal insulin secretion, may influence the child’s later pathology, contributing to a “developmental overnutrition” hypothesis [1,8]. Similarly, increased maternal adiposity before pregnancy is considered a risk factor; mothers classified as obese (BMI ≥ 30 kg/m^2^) had children with a higher risk of T1D (HR 1.22; 95% CI 1.11, 1.34) compared to those of normal weight in a study using Swedish registry data [9]. Furthermore, maternal BMI and neonatal birth weight were found to be the two most significant factors in models predicting neonatal blood glucose concentrations in infants born to mothers with gestational diabetes mellitus [10].

Results from studies examining infant growth patterns also provide key insights. Increased birth weight is positively associated with an increased risk of childhood-onset T1D [11]. A large meta-analysis found that high birth weight (≥4000 g) was associated with an elevated risk of T1D compared with the reference category (3.0–3.5 kg), demonstrating an adjusted OR of 1.11 (95% CI 1.03, 1.20) [12]. Conversely, low birth weight (<2.5 kg) often showed a non-significant or reduced risk compared to the reference range [12]. Correspondingly, children born Large for Gestational Age (LGA) are more often affected by T1D (4.7% of cases compared with 3.5% of controls in a Swedish cohort), while being born Small for Gestational Age (SGA) is less common among T1D cases (2.0% of cases compared with 2.6% of controls) [9]. A Mendelian randomization study suggested that a genetically predicted increase in childhood BMI was associated with a 32% increased risk of T1D (OR 1.32, 95% CI 1.06–1.64) [3].

Early postnatal exposures, particularly feeding and delivery mode, have also been investigated. Infant feeding studies suggest that never being fed human milk (breastfed) is associated with a higher risk of T1D [13]. A large study pooling data from Norwegian and Danish cohorts found that children who were never breastfed had a twofold increased risk of developing T1D compared to those who were ever breastfed [14]. While the evidence base is limited, some studies suggest that a longer duration of total or exclusive human milk feeding may confer protection. However, findings regarding the duration of breastfeeding and T1D risk are inconsistent across observational studies, potentially due to differences in statistical power, as T1D is a low-incidence outcome [13]. Regarding mode of delivery, C-section has been studied as a potential risk factor [2,15]. Although an unadjusted analysis in Finland showed children born by any cesarean section were at increased risk (HR = 1.17; 95% CI 1.09, 1.24) compared with vaginal delivery, this association was diminished after adjustment for various maternal characteristics [2]. However, studies consistently show that cesarean delivery significantly decreases the likelihood of timely initiation of breastfeeding, and the mode of delivery can influence the infant’s gut microbiota composition, which may, in turn, play a mechanistic role in autoimmunity development [2,16].

In this context, this research aims to evaluate the complex relationship between several neonatal and perinatal factors, specifically the Apgar score, birth weight, type of infant feeding, sex, and type of delivery, and the subsequent risk of developing Type 1 Diabetes Mellitus (T1D), by using correlations, general linear model and artificial neural network. Secondary aims include identifying the most powerful neonatal/perinatal predictors of T1D and exploring potential interactions between these investigated factors. This research is among the few to comprehensively evaluate multiple neonatal and perinatal factors in relation to the later development of Type 1 Diabetes Mellitus. Unlike previous studies focusing on individual or maternal predictors, this work investigates potential interactions among these early-life parameters and identifies the most powerful neonatal predictors of T1D risk. By integrating diverse perinatal variables into a single analytical model, this study aims to provide new insight into the early-life determinants of autoimmune diabetes.

## 2. Materials and Methods

### 2.1. Data Collection

The study employed an observational, retrospective design, structured as a cohort study, utilizing existing data extracted from medical registers, neonatal, and pediatric records between 2010 and 2025. The case group comprised children diagnosed with Type 1 Diabetes Mellitus (T1D) before the age of 18, while the control group consisted of children without T1D. The relationship between T1D (the dependent variable) and several perinatal predictors (independent variables) including Apgar score, categorized birth weight (Sga—small for gestational age, Aga—appropriate for gestational age, Lga—large for gestational age), neonatal feeding type (breast milk vs. formula), and type of delivery (vaginal vs. cesarean section) will be investigated. Sga refers to infants with a birth weight below the 10th percentile for their gestational age, Aga includes those between the 10th and 90th percentiles, and Lga describes infants above the 90th percentile. Exclusion criteria included: diagnosis with other forms of diabetes (e.g., Type 2 Diabetes), incomplete data for the variables of interest. In this context, the cohort comprised 327 patients.

The characteristics of the cohort are presented in Table 1. The control group accounted for 45.6% of the cohort, while T1D group represented 54.4%. Most of the patients were appropriate for the gestational age (Aga). A percent of 47.1% of the cohort was breastfed, while 52.9% received milk formula. A balanced proportion of men (51.7%) and women (48.3%) patients were included in the study. The natural vaginal birth represented 38.2%, while cesarean section accounted for 61.8%.

### 2.2. Statistical Data Processing

Comparisons between groups were performed using Mann–Whitney U test. The significance level considered was 95%. Spearman correlation and Principal Component Analysis (PCA) with Varimax rotation were employed to study the relationships between variables. General Linear Model (GLM) was used to investigate the effect of factors (birth weight, nutrition type and Apgar score) and their interaction on Diabetes risk. Model suitability was evaluated through Fisher F test, along with R^2^ and Adj.-R^2^ values, while the significance of factors was evaluated through Fisher test (*p* < 0.05 was considered significant).

All analyses and model training were performed using IBM SPSS Statistics (trial version) on a computer (ASUS, ASUSTeK Computer Inc., Taipei, Taiwan) equipped with an Intel^®^ Core™ i7-1065G7 CPU @ 1.30 GHz, 4 GB RAM, and a 238 GB SSD. The device uses Intel^®^ Iris^®^ Plus Graphics (128 MB). The default SPSS random seed settings were fixed to maintain consistency across runs. All preprocessing, model building, and validation steps were conducted within SPSS. To ensure consistency, all ANN analyses were repeated multiple times, and the results were found to be stable.

### 2.3. Artificial Neural Network Architecture

The Artificial Neural Network (ANN) served as a powerful machine learning complement to the traditional statistical analysis, offering a non-linear approach crucial for complex biological phenomena like diabetes risk. Its primary advantage is the ability to automatically detect and model intricate, non-linear interactions among predictor variables, such as nutrition type, birth weight, and Apgar score, without requiring these interactions to be manually defined as in the GLM. The main disadvantage of the ANN is its limited interpretability of the results, making it difficult to extract the magnitude and direction of individual factor influence, unlike the GLM. The ANN was used primarily to capture and model the non-linear, complex interactions inherent in early-life diabetes risk. Together with GLM, which offers more specific impact of each factor, this approach can provide a comprehensive and robust understanding of diabetes risk.

All continuous variables were standardized prior to analysis, and categorical variables were encoded using one-hot encoding to ensure equal weighting in the ANN model. Predicting various diseases, including diabetes and its complications, relies heavily on machine learning models. Artificial Neural Networks (ANN) are especially important because they can learn complex patterns in medical data, enabling more accurate and early disease prediction. Artificial Neural Network was used for Diabetes risk prediction in function of birth weight, nutrition type, and Apgar score as factors, along with sex and delivery type as covariate factors. Partition of the dataset was as follows: 70% for training and 30% for testing. The minimum number of units in the hidden layer was set to 1, and the maximum to 50. The type of training was Batch, and the optimization algorithm was the scaled conjugate gradient. The dataset was split using a hold-out method, with 225 cases (74.8%) assigned to the training set and 76 cases (25.2%) assigned to the testing set, and 26 cases were excluded. ANN model performance was evaluated using Receiver Operating Characteristic (ROC) analysis, Kappa, standard error of mean (SME), and root mean squared deviation (RMSE) [17] (Equations (1)–(6)).

(1) $$Accuracy\;(\%)=\frac{TP+TN}{FP+FN+TP+TN}\times 100$$

(2) $$Sensitivity\left(\%\right)=\frac{TP}{FN+TP}\times 100$$

(3) $$Specificity\left(\%\right)=\frac{TN}{FP+TN}\times 100$$

(4) $$AUC=\sum_{i=1}^{n-1}\sum ({FPR}_{i+1}-{FPR}_{i})\times \frac{{TPR}_{i+1}+{TPR}_{i}}{2}$$

where

(5) $$TPR=\frac{TP}{FN+TP}$$

(6) $$FPR=\frac{FP}{TN+FP}$$

- *TP* = True Positives;
- *TN* = True Negatives;
- *FP* = False Positives;
- *FN* = False Negatives.

The artificial neural network was developed to predict Type 1 Diabetes Mellitus risk, using five predictor variables: nutrition type, birth weight, Apgar score, sex, and delivery type. Categorical predictors were expanded into a total of 99 input units after encoding, and all covariates were standardized to ensure comparable scaling and improve model convergence (Table 2). The network contained a single hidden layer comprising three neurons, which employed the hyperbolic tangent activation function to capture non-linear relationships among the predictors. The output layer consisted of two units corresponding to the two diabetes categories, using the Softmax activation function to generate predicted probabilities. This architecture was selected based on preliminary experiments and the dataset size, aiming to balance performance and avoid overfitting. To select the network architecture, systematic exploratory experiments were performed varying the number of hidden neurons (1–50) and activation functions (identity, hyperbolic tangent, sigmoid). These tests demonstrated that a single hidden layer with three neurons using the hyperbolic tangent activation function provided the best balance between predictive performance and model simplicity. Early-stopping criteria were also evaluated, confirming that the chosen architecture remained robust. According to the data presented in Table S3 (Supplementary Material), the use of different activation functions (identity, hyperbolic tangent, sigmoid) resulted in lower AUC values (0.54–0.94) compared to the model selected which uses softmax activation function (AUC = 0.95). In addition, the increase in the number of hidden layers and stopping rule (3 or 5 consecutive steps with no decrease in error) also generated models with lower AUC (0.90–0.91). Overall, these experiments informed the final design and justify the selected configuration by explicitly linking model behavior and dataset characteristics to the architectural choices (Table S3, Supplementary Material).

## 3. Results

### 3.1. Comparisons Between Groups

According to the results displayed in Table 3, T1D group presented higher birth weight class and birth weight, and lower Apgar score compared to the control group. In T1D group the predominant nutrition type was formula, and the delivery type was mostly cesarean section (Table 3). Mann–Whitney test revealed significant differences (*p* < 0.05) between control and T1D group for the characteristics considered, except for sex.

### 3.2. Relationships Between Variables

Spearman correlations were used to highlight the relationships between variables (Table 4). Birth weight and birth weight class were weakly positively correlated with nutrition type (r > 0.34, *p* < 0.05). Diabetes risk was moderately positively correlated with birth weight class and nutrition type (r > 0.40, *p* < 0.05), while with Apgar score the correlation was negative and weak (r = −0.37, *p* < 0.05). According to our results, lower Apgar score led to higher diabetes risk. Delivery type was weakly positively correlated with birth weight, birth weight class and nutrition type (0.30 < r < 0.39, *p* < 0.05). In particular, cesarean section may be related to formula-nutrition type and higher birth weight.

**Figure 1:**
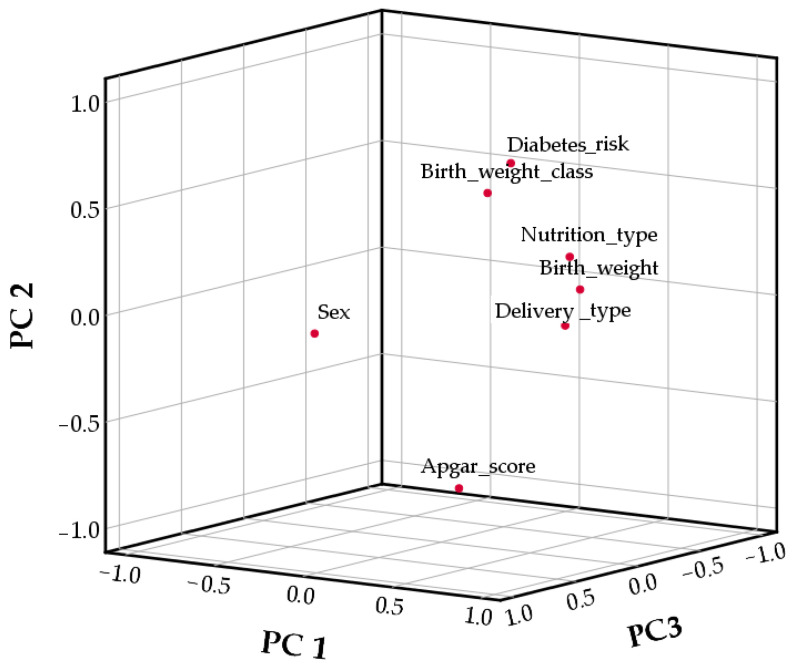
Principal component Analysis graphic.

For further investigation of the relationships between variables, Principal Component Analysis was used (Table 5, Figure 1). The adequacy of the dataset for factor analysis was evaluated using the Kaiser–Meyer–Olkin (KMO) Measure of Sampling Adequacy and Bartlett’s Test of Sphericity. The KMO value of 0.73 exceeded the recommended threshold of 0.60, indicating that the sample size and the correlations among variables were adequate for reliable factor extraction. Bartlett’s Test of Sphericity produced a Chi-square value of 303.23 (df = 21, *p* < 0.001), confirming that the variables shared sufficient common variance. These results demonstrate that the data were suitable for factor analysis and support the validity of conducting further component extraction. According to Table 5, PC1 explained 33.93% of data variability, followed by PC2, which explained 15.60% of the total variance, and PC3, which accounted for 14.41%.

The first principal component (PC1) was associated with nutrition type, birth weight, and delivery type (Figure 1, Table S1—Supplementary Material). PC2 was associated with birth weight class, Apgar score, and diabetes risk, while PC3 was associated only with sex.

### 3.3. Multivariate Analysis and Artificial Neural Network for T1D

The results of the General Linear Model (Table 6) analysis showed that the overall model was statistically significant in predicting Diabetes risk (F = 4.55, *p* < 0.01), explaining approximately 88% of the variance (R^2^ = 0.88, Adjusted R^2^ = 0.69).

The intercept was highly significant (F = 145.27, *p* < 0.01, η_p_^2^ = 0.54), confirming a strong overall model fit. Among the main effects, Nutrition type significantly influenced Diabetes type (F = 4.04, *p* = 0.045, η^2^ = 0.03), while Birth weight showed a highly significant effect (F = 3.65, *p* < 0.01, η_p_^2^ = 0.76), suggesting that variations in birth weight are strongly associated with diabetes outcomes. Apgar score also had a significant effect (F = 3.98, *p* < 0.01, η_p_^2^ = 0.16), indicating its predictive contribution. Regarding interactions, the Nutrition type × Birth weight interaction was significant (F = 2.93, *p* < 0.01, η_p_^2^ = 0.31), demonstrating that the relationship between birth weight and diabetes type varies by nutritional status. The Nutrition type × Apgar score interaction was also significant (F = 3.55, *p* = 0.03, η_p_^2^ = 0.05), implying that the influence of Apgar score on diabetes type depends partially on nutrition type. However, the Birth weight × Apgar score (F = 1.32, *p* = 0.12) and the three-factors interaction (Nutrition type × Birth weight × Apgar score, F = 0.80, *p* = 0.49) were not statistically significant, indicating that the combined effect of all three variables does not add further predictive value.

ANN model performance was optimized using the cross-entropy error function. During training, the model achieved a cross-entropy error of 58.01, while testing on unseen data revealed a cross-entropy error of 29.15 (Table 7). The percentage of incorrect predictions was 7.10% during training and 15.8% during testing. Overall, these results indicate that the model generalizes well to new data.

**Figure 2:**
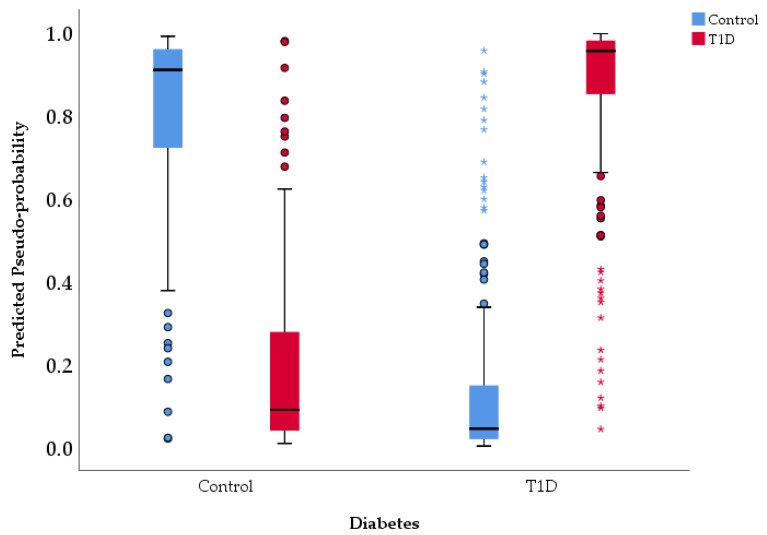
Predicted vs. observed values chart.

The graphical representation of predicted vs. observed values for Diabetes variable is presented in Figure 2.

The artificial neural network (ANN) demonstrated robust predictive performance in classifying patients with T1D (Table 8 and Table S2—Supplementary Material). Based on the testing set, the model correctly identified 41 true positive (T1D) cases and 23 true negative (control) cases, with 7 control cases misclassified as T1D (false positives) and 5 T1D cases misclassified as control (false negatives). This corresponds to an overall accuracy of 84.2%, indicating that the model correctly classified the majority of cases in the testing set.

**Figure 3:**
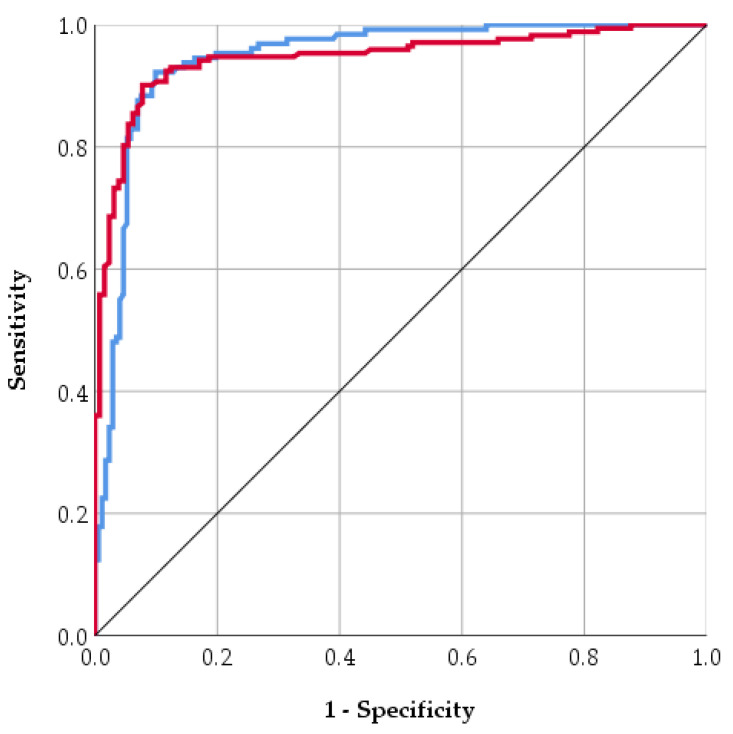
ROC curve for control (blue line) and T1D (red line).

The model showed strong discriminative ability, with a sensitivity (recall) of 89.1% for T1D, meaning it correctly identified most T1D cases, and a specificity of 76.7% for Control, reflecting its ability to correctly classify non-diabetic cases. The positive predictive value (PPV) of 85.4% indicates that predicted T1D cases were highly likely to be true positives, while the negative predictive value (NPV) of 82.1% suggests good reliability in identifying controls. Additional performance metrics further support the model’s robustness. The area under the curve (AUC) of 0.95 indicates good discrimination between T1D and Control cases. The Kappa statistic of 0.81 reflects good agreement between predicted and observed classifications, beyond chance. Prediction errors were low, with a mean absolute error (MAE) of 0.09 and a root mean squared error (RMSE) of 0.31, demonstrating that predicted probabilities were generally close to the true labels. Discrimination between classes was also strong, with an area under the ROC curve (AUC) of 0.95, suggesting that the model can effectively distinguish between T1D and control cases (Figure 3). Figure 3 displays the trade-off between Sensitivity and Specificity for control and T1D. Both curves are located tightly toward the top-left corner, far from the line of chance, confirming the good discriminative ability of the model (consistent with the reported AUC of 0.95). Specifically, the curves demonstrate that the model can achieve a very high Sensitivity (near 90%) while maintaining a low False Positive Rate (below 20%), although the blue curve (control) is marginally closer to the optimal corner, suggesting slightly better separation or classification confidence for the control group data compared to the T1D group data. Error metrics show that the average deviation of predicted probabilities from actual labels was relatively low (MAE = 0.09), though RMSE (0.31) indicates that a small subset of predictions deviated more substantially from true labels. Overall, these results indicate that the ANN provides robust, accurate, and reliable predictions of diabetes type, with strong discriminatory ability and high agreement with observed outcomes. The low number of False Negatives (FN = 5), supported by the 89.1% sensitivity, is the model’s key clinical advantage, as it minimizes the severe risk of overlooking a truly high-risk patient. This, combined with the high Positive Predictive Value (PPV) of 85.4%, validates the model’s reliability in flagging patients. The strong performance metrics, especially the AUC of 0.95 and low prediction errors, suggest that the non-linear synergy among factors like nutrition type, birth weight, and Apgar score forms a potent and measurable early-life risk signature.

## 4. Discussion

Type 1 Diabetes Mellitus (T1D) is an autoimmune disorder characterized by the destruction of beta-cells, leading to insulin deficiency and subsequent hyperglycemia in genetically predisposed individuals. While genetics is a major component, disease development is substantially influenced by the interaction between the immune system and environmental factors [18]. The results obtained through the artificial neural network (ANN) model in this study suggest that perinatal and neonatal data can provide valuable information for estimating the risk of developing Type 1 Diabetes Mellitus (T1D). The finding that high birth weight and cesarean delivery are associated with an increased risk of T1D is consistent with previous large population-based studies from Sweden and Finland, which have reported similar but modest effects [2,9]. A study demonstrated that the increasing trends in birthweight and childhood overweight in Sweden parallel the rising incidence of early-onset Type 1 Diabetes Mellitus [19]. Another paper indicated that birth weight > 4000 g increases the risk of T1D by 43% [20]. Increased birth weight and birth weight for gestational age are associated with a modestly elevated risk of developing T1D, as previously reported by Haynes et al. [11]. The mechanism by which the intrauterine environment influences T1D risk is unclear, but the accelerator hypothesis proposes that chronic beta-cell secretory demand, possibly due to factors like overnutrition or obesity, activates intrinsic stress pathways that trigger or accelerate beta-cell autoimmunity [19,21]. Elevated intrauterine glucose exposure in LGA neonates stimulates increased fetal beta-cell insulin secretion, which acts as a major fetal growth factor [1,12,20]. This heightened beta-cell activity may render them more susceptible to autoimmune destruction, linking LGA status to increased risk for Type 1 Diabetes Mellitus and subsequent childhood obesity, an effect that may also involve metabolic imprinting across generations [3,22,23].

Our results indicated a relationship between delivery type and nutrition: cesarean section led to lower chance of breastfeeding. Cesarean delivery is associated with significantly reduced odds of timely breastfeeding initiation (79% lower odds compared to vaginal delivery in a large meta-analysis), likely due to physiological and procedural factors such as maternal recovery, anesthesia effects, and mother-infant separation [24]. Infants born via C-section are more likely to receive early formula supplementation. A study by Liu et al. [25] reported that women with planned cesarean deliveries had a higher formula feeding rate (2.1%) and a lower exclusive breastfeeding rate (89.7%) compared to those with planned vaginal deliveries (1.0% and 92.4%, respectively). T1D risk may be associated with delivery type and nutrition. Research found that formula-fed or cesarean-delivered infants had different trajectories of gut bacterial colonization compared to vaginally born and breastfed infants, which could have implications for their future health [16,26]. A study found that children who were never breastfed had a twofold increased risk of developing T1D compared to those who were breastfed [14]. However, another one suggested that limited to moderate evidence indicates that feeding less or no human milk is associated with a higher risk of T1D in offspring [13]. Caicedo et al. [18] observed that formula feeding during the neonatal period may influence the development of T1D in susceptible individuals through an intestinal mucosal inflammatory response, resulting in loss of self-tolerance. Contrary to our results, a study in Scotland reported no statistically significant association observed between a child’s Apgar score and the subsequent development of Type 1 Diabetes Mellitus [6]. This difference could be due to the fact that T1D risk is strongly influenced by Human Leukocyte Antigen genotypes [5], so populations with different genetic backgrounds might show varying susceptibility.

The performance of the ANN model, expressed by an Area Under the Curve (AUC) value higher than that of logistic regression models, demonstrates the advantage of applying artificial intelligence techniques to complex medical datasets. Neural networks can identify non-linear interactions among variables, suggesting that T1D susceptibility is shaped not only by genetic or autoimmune determinants but also by early-life metabolic and inflammatory contexts. Utilizing maternal and neonatal clinical data, a study reported that the ANN model accurately predicted neonatal blood glucose levels with a high R^2^ of 0.869 and an RMSE of 0.274, with neonatal birth weight and maternal BMI being the most significant predictors of hypoglycemia [10].

Both the General Linear Model (GLM) and Artificial Neural Network (ANN) effectively predicted diabetes risk using early-life factors. The GLM explained approximately 88% of the variance and provided interpretable effects of predictors and their interactions, highlighting the contribution of birth weight, Apgar score, and nutrition type. The ANN, however, achieved higher predictive performance (testing accuracy 84.2%, AUC 0.946, Kappa 0.81) and captured complex non-linear relationships that the GLM may not fully detect. While GLM offers insight into variable effects, the ANN provides a robust tool for early identification of at-risk individuals, suggesting that combining both approaches could enhance predictive power and clinical understanding.

Nevertheless, several limitations should be acknowledged. First, the retrospective nature of the data may introduce biases due to incomplete or inconsistent documentation of key variables. Additionally, some important risk factors for Type 1 Diabetes Mellitus, including genetic predisposition and environmental exposures, were not included, which may limit the comprehensiveness of the models. The findings were derived from a single dataset without external validation, which may restrict the generalizability of the results to broader populations. Relying solely on perinatal and neonatal data does not provide a comprehensive risk estimate, as genetic factors (such as HLA haplotypes) and specific autoantibodies play major roles in T1D pathogenesis. Second, the analyzed data may be subject to registry bias or inconsistencies in the clinical definitions of variables (e.g., birth weight, gestational duration). Additionally, the single-center design may limit applicability to broader populations with differing genetic or socio-environmental profiles. Another limitation of this study is that only a single ANN architecture was evaluated, which restricts our ability to draw definitive conclusions about the suitability or superiority of ANNs compared to alternative models or architectures. Furthermore, the training stopping criterion was set to terminate after only one consecutive step without error improvement, which may lead to premature stopping and convergence to suboptimal solutions. The study’s high-performance metrics, in the context of a small dataset without external validation, could indicate a potential for overfitting and caution in generalizing the results.

Despite these limitations, this study highlights the real potential of ANN-based models in predictive medicine. Integrating such approaches with genetic, immunologic, and environmental information could lead to the development of a multimodal prediction model for T1D risk, applicable in neonatal screening programs. In the future, these tools could support the personalization of preventive interventions and the optimization of early monitoring strategies for children at increased risk.

## 5. Conclusions

This study demonstrates that an artificial neural network (ANN) and General Linear Model (GLM) can effectively predict Type 1 Diabetes Mellitus (T1D) using early-life factors such as birth weight, Apgar score, nutrition type, sex, and delivery type. The GLM successfully predicted diabetes risk (*p* < 0.01) and accounted for a high variance (88%), with significant interaction effects among nutrition type, birth weight, and Apgar score. Although each factor shows only moderate-to-weak correlations individually, their combination in the ANN model yielded high predictive accuracy (84.2%), sensitivity (89.1%), and AUC (0.95). However, this model should be considered a proof-of-concept, as it has not yet been validated externally, and does not include genetic data or autoimmune markers. These findings require validation using larger, independent datasets. This approach could contribute to early identification and targeted interventions for at-risk individuals. Importantly, the integration of ANN-based risk estimation into clinical workflows could support neonatologists and pediatric endocrinologists in identifying children who may benefit from closer metabolic monitoring or early preventive counseling. Such predictive tools could be further incorporated into electronic health record (EHR) systems, enabling automated flagging of high-risk profiles at the moment of birth or during early postnatal follow-up. Future research should aim to externally validate this model in multi-center cohorts and assess its performance across populations with diverse genetic backgrounds. In addition, combining clinical neonatal data with immunological (e.g., autoantibody status) and genomic markers (e.g., HLA haplotypes) may further enhance predictive accuracy and support the development of a comprehensive early-life risk stratification tool for T1D. Ultimately, this work contributes to the foundation for precision prevention approaches in childhood diabetes, emphasizing the potential to shift from reactive clinical care to proactive risk-guided monitoring and intervention.

## Figures and Tables

**Table 1:** Characteristics of the cohort.

Characteristic	Frequency	Percent (%)
***Birth weight class***		
Aga	226	69.1
Lga	82	25.1
Sga	19	5.8
***Nutrition type***		
Breast milk	154	47.1
Formula	173	52.9
***Sex***		
Men	169	51.7
Women	158	48.3
***Delivery type***		
Vaginal	125	38.2
Cesarean section	202	61.8
***Diabetes risk***		
Control	149	45.6
T1D	178	54.4

Aga—appropriate for gestational age, Lga—large for gestational age, Sga—small for gestational age, T1D—Type 1 Diabetes Mellitus.

**Table 2:** Artificial Neural Network architecture.

**Input Layer**	Factors	1	Nutrition type
		2	Birth weight
		3	Apgar score
	Covariates	1	Sex
		2	Delivery type
	Number of Units		99
	Rescaling Method for Covariates		Standardized
**Hidden Layer(s)**	Number of Hidden Layers		1
	Number of Units in Hidden Layer 1		3
	Activation Function		Hyperbolic tangent
**Output Layer**	Dependent Variable		Diabetes risk
	Number of Units		2
	Activation Function		Softmax
	Error Function		Cross-entropy
**Training time**			0.15 s
**Inference time**			0.003 s

**Table 3:** Comparison between groups.

	Diabetes Type	Birth Weight Class	Nutrition Type	Birth Weight	Sex	Apgar Score	Delivery Type
Mean	Control	1.14	1.30	3313.76	1.49	8.80	1.52
	T1D	1.56	1.72	3828.31	1.48	8.04	1.70
Std. Deviation	Control	0.44	0.46	473.84	0.50	0.89	0.50
	T1D	0.64	0.45	912.14	0.50	1.07	0.46
Median	Control	1.00	1.00	3280.00	1.00	9.00	2.00
	T1D	1.00	2.00	3800.00	1.00	8.00	2.00
Std. Error of Mean	Control	0.04	0.04	38.82	0.04	0.07	0.04
	T1D	0.05	0.03	68.37	0.04	0.08	0.03
Mann–Whitney U test (*p*-value)		0.00	0.00	0.00	0.91	0.00	0.00

T1D—Type 1 Diabetes Mellitus.

**Table 4:** Relationships between variables expressed by Spearman correlations.

	Birth Weight Class	Nutrition Type	Birth Weight	Sex	Apgar Score	Delivery Type	Diabetes Risk
Birth weight class	1.00						
Nutrition type	0.34 **	1.00					
Birth weight	0.42 **	0.36 **	1.00				
Sex	0.01	−0.02	−0.06	1.00			
Apgar score	−0.27 **	−0.19 **	−0.12 *	0.01	1.00		
Delivery type	0.30 **	0.39 **	0.32 **	0.02	−0.08	1.00	
Diabetes risk	0.40 **	0.43 **	0.34 **	−0.01	−0.37 **	0.19 **	1.00

**—correlation is significant at the 0.01 level (2-tailed), *—correlation is significant at the 0.05 level (2-tailed).

**Table 5:** Eigenvalues and data variance explained by the principal components (PC).

Component	Initial Eigenvalues	% of Variance	Cumulative %
PC1	2.38	33.93	33.93
PC2	1.09	15.60	49.54
PC3	1.01	14.41	63.94
Kaiser–Meyer–Olkin Measure of Sampling Adequacy		0.73	
Bartlett’s Test of Sphericity	Approx. χ^2^	303.23	
	df	21	
	*p*-value	0.00	

**Table 6:** General linear model results showing the effects of nutrition type, birth weight, and Apgar score on T1D diabetes risk.

Source	Type III SS	df	MS	F	*p*-Value	η_p_^2^
Corrected Model	71.36	201.00	0.36	4.55	0.00	0.88
Intercept	11.33	1.00	11.33	145.27	0.00	0.54
Nutrition type	0.32	1.00	0.32	4.04	0.05	0.03
Birth weight	31.03	109.00	0.28	3.65	0.00	0.76
Apgar score	1.86	6.00	0.31	3.98	0.00	0.16
Nutrition type × Birth weight	4.34	19.00	0.23	2.93	0.00	0.31
Nutrition type × Apgar score	0.55	2.00	0.28	3.55	0.03	0.05
Birth weight × Apgar score	4.74	46.00	0.10	1.32	0.12	0.33
Nutrition type × Birth weight × Apgar score	0.19	3.00	0.06	0.80	0.49	0.02

R^2^ = 0.88 (Adjusted R^2^ = 0.69), SS—sum of square, MS—mean square, η_p_^2^—partial eta squared.

**Table 7:** Model summary.

**Training**	Cross-Entropy Error	58.01
	Percent Incorrect Predictions	7.10%
	Stopping Rule Used	1 consecutive step(s) with no decrease in error ^a^
**Testing**	Cross-Entropy Error	29.15
	Percent Incorrect Predictions	15.8%

Dependent variable: Diabetes risk. ^a^ Error computations are based on the testing sample.

**Table 8:** Performance parameters of ANN.

Metric	Value
True Positive (TP)	41
True Negative (TN)	23
False Positive (FP)	7
False Negative (FN)	5
Accuracy	84.2%
Sensitivity (Recall/True Positive Rate)	89.1%
Specificity (True Negative Rate)	76.7%
Positive Predictive Value (PPV/Precision)	85.4%
Negative Predictive Value (NPV)	82.1%
Area Under the Curve (AUC)	0.95
Kappa	0.81
Mean Absolute Error (MAE)	0.09
Root Mean Squared Error (RMSE)	0.31

## Data Availability

The data are available upon request at the corresponding author.

## Supplementary Materials

The following supporting information can be downloaded at: https://www.mdpi.com/article/10.3390/life15121800/s1. Table S1. Rotated Component Matrix; Table S2. Classification table; Table S3. Other ANN models networks.
