# Supplementary material for: Neonatal and Birth Risk Factors for Type 1 Diabetes Mellitus: Prediction Using an Artificial Neural Network
Source: Life (Basel). 2025 Nov 24;15(12):1800. doi: 10.3390/life15121800 (PMC12733860; doi:10.3390/life15121800)
Supplement: Supplementary file 1 [file life-15-01800-s001.zip › life-3989542-supplementary.pdf]

SUPPLEMENTARY MATERIAL

Table S1. Rotated Component Matrix[a]

Variable	Component PC1	PC2	PC3
Birth weight class	0.326	0.573	0.092
Nutrition type	0.711	0.296	-0.018
Birth weight	0.700	0.128	-0.116
Sex	-0.021	0.008	0.987
Apgar score	0.127	-0.840	0.037
Birth type	0.768	-0.003	0.101
Diabetes risk	0.364	0.697	-0.039

Extraction Method: Principal Component Analysis.
Rotation Method: Varimax with Kaiser Normalization.
a. Rotation converged in 4 iterations.

Table S2. Classification table

Sample	Observed	Predicted Control	T1D	Percent Correct
Training	Control	91	8	91.9%
	T1D	8	118	93.7%
	Overall Percent	44.0%	56.0%	92.9%
Testing	Control	23	7	76.7%
	T1D	5	41	89.1%
	Overall Percent	36.8%	63.2%	84.2%

Dependent Variable: Diabetes

Table S3. Other ANN models networks

	Model	Model 1	Model 2	Model 3	Model 4	Model 5	Model 6
Input Layer	Factors	Nutrition type<br>Birth weight<br>Apgar score	Nutrition type<br>Birth weight<br>Apgar score	Nutrition type<br>Birth weight<br>Apgar score	Nutrition type<br>Birth weight<br>Apgar score	Nutrition type<br>Birth weight<br>Apgar score	Nutrition type<br>Birth weight<br>Apgar score
	Covariates	Sex<br>Delivery type	Sex<br>Delivery type	Sex<br>Delivery type	Sex<br>Delivery type	Sex<br>Delivery type	Sex<br>Delivery type
	Number of Units	100	106	100	100	102	102
	Rescaling Method for Covariates	Standardized	Standardized	Standardized	Standardized	Standardized	Standardized
Hidden Layer(s)	Number of Hidden Layers	2	2	2	2	1	1
	Number of Units in Hidden Layer	2	2	2	5	12	12
Output Layer	Activation function	Sigmoid	Sigmoid	Sigmoid	Hyperbolic tangent	Hyperbolic tangent	Hyperbolic tangent
	Dependent Variable	Diabetes_type	Diabetes_type	Diabetes_type	Diabetes_type	Diabetes_type	Diabetes_type
	Number of units	2	2	2	2	2	2
	Activation function	Identity	Hyperbolic tangent	Sigmoid	Softmax	Softmax	Softmax
	Error function	Sum of squares	Sum of squares	Sum of squares	Cross-entropy	Cross-entropy	Cross-entropy
Training	Sum of Squares Error	55.450	30.837	20.237	70.146	86.598	83.589
	Percent Incorrect Predictions	44.0%	16.1%	11.3%	14.5%	17.7%	12.9%
	Stopping Rule Used	1 consecutive step(s) with no decrease in error	1 consecutive step(s) with no decrease in error	1 consecutive step(s) with no decrease in error	1 consecutive step(s) with no decrease in error	3 consecutive step(s) with no decrease in error	5 consecutive step(s) with no decrease in error
	Training Time	0:00:00.02	0:00:00.11	0:00:00.12	0:00:00.13	0:00:00.23	0:00:00.11
Testing	Sum of Squares Error	18.246	9.915	7.535	22.639	29.826	28.683
	Percent Incorrect Predictions	39.5%	23.2%	11.8%	15.5%	17.3%	15.7%
	**AUC**	**0.54**	**0.89**	**0.94**	**0.94**	**0.90**	**0.91**
